# The Seattle Angina Questionnaire and Quality of Life in Chronic Coronary Syndrome: Opportunities for Implementation in Bulgarian Clinical Practice—A Narrative Review

**DOI:** 10.3390/medicina61111924

**Published:** 2025-10-27

**Authors:** Velina Doktorova, Georgi Goranov, Petar Nikolov

**Affiliations:** 1 First Department of Internal Diseases, Section of Cardiology, Medical University of Plovdiv, 4000 Plovdiv, Bulgaria; 2 Department of Interventional Cardiology, University Multiprofile Hospital for Active Treatmen “Sveti Georgi”, 4000 Plovdiv, Bulgaria; 3 Department of Cardiovascular Surgery, Medical University of Plovdiv, 4000 Plovdiv, Bulgaria

**Keywords:** SAQ, CCS, ANOCA/INOCA, microvascular angina, vasospastic angina, PRO, Bulgaria, pathway, CFR, IMR, acetylcholine testing, PCI

## Abstract

*Background*: Patient-reported outcomes are integral to chronic coronary syndrome (CCS) care. The Seattle Angina Questionnaire (SAQ) is validated and prognostic, yet its clinical integration in Bulgaria is undefined. Aim: The aim of this study was to provide a structured, clinically oriented framework for integrating the SAQ into the full CCS care pathway—from screening and phenotyping (obstructive vs. ANOCA/INOCA endotypes) to diagnostics, mechanism-tailored therapy, and follow-up—while outlining a pragmatic roadmap for Bulgarian implementation. *Methods*: We conducted a semi-structured narrative review (1995–2024) of SAQ’s validation, prognostic utility, and implementation in the literature, augmented with guideline-based frameworks for CCS/ANOCA care. *Results*: The SAQ (and SAQ-7) shows strong reliability and responsiveness and independently predicts health status and clinical outcomes. Embedding the SAQ at baseline, at 4–12 weeks after therapy changes, and after 6–12 months enables symptom-guided decision-making. A phenotype-guided pathway is proposed that couples the SAQ with CAD burden assessment and—where indicated—ANOCA diagnostics (CFR/IMR, vasoreactivity testing). Mechanism-tailored therapy maps to endotypes (e.g., VSA → CCB ± nitrates; MVA → beta-blocker/ACEi/statin ± ranolazine; obstructive CADGDMT ± PCI/CABG). A minimum dataset, metrics, and registry fields are specified for Bulgarian deployment. *Conclusions*: A clinically structured framework clarifies how the SAQ adds value beyond description—by informing triage, treatment selection, and follow-up across CCS phenotypes. This approach provides educational guidance and a practical blueprint for pilot implementation in Bulgaria.

## 1. Introduction

Cardiovascular disease remains the leading cause of morbidity and mortality worldwide, with chronic forms of coronary artery disease posing a particularly significant burden [1,2]. For decades, the clinical entity traditionally referred to as stable ischemic heart disease (SIHD) represented the main focus of outpatient management in patients with angina. However, mounting evidence has demonstrated that coronary artery disease is rarely static. Instead, it reflects a dynamic process of plaque progression, endothelial dysfunction, and microvascular impairment, which results in fluctuations in symptoms and prognosis over time.

Reflecting this shift in understanding, the 2019 European Society of Cardiology (ESC) Guidelines introduced the term chronic coronary syndromes (CCSs) to replace “stable ischemic heart disease”, recognizing the broader and more dynamic spectrum of the condition [1]. This paradigm has been reinforced in the 2024 ESC update on CCSs [3], which further expanded the concept to include patients with angina and/or ischemia without obstructive coronary disease (ANOCA/INOCA). Parallel to this, the 2023 AHA/ACC Guideline for the Management of Patients with Chronic Coronary Disease (CCD) [4] retired the SIHD terminology and endorsed a comprehensive framework that better reflects the diversity of chronic coronary presentations. Both guidelines emphasize a patient-centered approach, shared decision-making, and structured long-term management while highlighting advances in diagnostic strategies—including non-invasive functional imaging, invasive physiological testing, and the recognition of microvascular and vasomotor dysfunction—that enable more precise characterization of ischemia and support individualized therapy. These developments underscore the need for outcome measures that extend beyond survival or recurrent myocardial infarction, capturing patient symptoms and health-related quality of life as integral endpoints.

Despite these international advances, the use of patient-reported outcomes (PROs) in Bulgarian cardiology remains limited. Current national practice is primarily focused on traditional clinical endpoints, with less attention paid to the subjective dimensions of care such as quality of life, symptom burden, and treatment satisfaction. This gap is particularly concerning given Bulgaria’s persistently high cardiovascular mortality rates, which reflect not only biological disease burden but also systemic challenges, including limited access to specialized cardiology services, shortages in the healthcare workforce, and underfunded prevention programs [5,6,7]. Several structural barriers explain why Bulgaria lags behind in the adoption of patient-reported outcomes. At present, there are no national cardiovascular registries systematically capturing PRO data, and health information systems remain fragmented, with limited interoperability between hospitals and outpatient practices [5,6,8]. Reimbursement models focus almost exclusively on procedural and survival outcomes, leaving no incentives for the routine assessment of health status or quality of life. Moreover, digital integration in outpatient care is underdeveloped, with heterogeneous electronic record systems and minimal capacity for standardized data collection [6]. These systemic barriers not only hinder the routine use of PRO instruments such as the SAQ but also contribute indirectly to Bulgaria’s persistently high cardiovascular mortality by preventing comprehensive quality monitoring and patient-centered evaluation. In this context, the integration of validated PRO measures could serve as an important component of national quality improvement efforts, complementing existing clinical endpoints with outcomes that more directly reflect the patient perspective. Addressing these systemic issues requires comprehensive strategies. Incorporating validated PROs into clinical practice represents one feasible step toward aligning with contemporary international standards and ensuring that care addresses outcomes that matter most to patients.

The Seattle Angina Questionnaire (SAQ) emerges as a leading candidate to fill this role. As a disease-specific, validated PRO tool, the SAQ captures five domains—Physical Limitation, Angina Stability, Angina Frequency, Treatment Satisfaction, and Quality of Life—that together provide a nuanced picture of patient health status [9]. It has demonstrated prognostic value and responsiveness to therapeutic interventions in multiple large-scale international studies, including COURAGE, ISCHEMIA, and EuroCTO [10,11,12,13]. However, its applicability in Bulgaria remains unexplored, with no translation, cultural validation, or implementation studies available to date. The present review therefore aims to (i) summarize the evidence base supporting the SAQ, (ii) critically evaluate its potential role in Bulgaria, and (iii) discuss the contextual, cultural, organizational, and economic considerations that would need to be addressed for successful implementation.

Against this background, the purpose of this review is not only to summarize the evidence base on the Seattle Angina Questionnaire (SAQ) but also to provide a structured, clinically oriented framework for its integration across the management pathway of chronic coronary syndromes. Specifically, we aim to clarify how SAQ assessment can be embedded at key decision points—from initial screening and phenotyping (obstructive CAD vs. ANOCA/INOCA and their endotypes), through CAD burden assessment and targeted diagnostics, to mechanism-tailored therapy and longitudinal follow-up. By aligning SAQ use with contemporary ESC and AHA/ACC guideline frameworks, and by contextualizing feasibility within the Bulgarian healthcare system, we seek to deliver not only a descriptive overview but also an educational and practice-oriented roadmap for implementation.

## 2. Methods

A semi-structured literature search was conducted to identify publications related to the Seattle Angina Questionnaire (SAQ), its development, psychometric properties, prognostic value, and clinical implementation. Searches were performed in PubMed, Scopus, and Web of Science databases. The following key terms and combinations were used: “Seattle Angina Questionnaire”, “SAQ”, “patient-reported outcomes”, “chronic coronary syndrome”, “quality of life”, “angina”, and “Bulgaria.” Reference lists of selected articles were additionally screened to capture relevant studies not retrieved through the database search. This review was designed as a semi-structured narrative review rather than a full systematic review, and therefore did not follow PRISMA guidelines. The goal was not to exhaustively capture every publication but to summarize key developments in the validation, prognostic value, and clinical implementation of the SAQ, with particular attention paid to contextual factors relevant for Bulgaria

The search covered the period from 1995 (the year of the SAQ’s first description) through March 2024. Only peer-reviewed articles published in English were included. The exclusion criteria were as follows: (i) conference abstracts without full-text publications, (ii) non-peer-reviewed materials, and (iii) articles not reporting original data or reviews directly related to the SAQ.

Article screening and eligibility assessment were performed independently by two reviewers, with disagreements resolved through consensus discussion. This approach was adopted to enhance reproducibility and reduce selection bias.

We recognize that the exclusion of non-English studies represents a limitation of this review, as it may have led to the omission of regional or local evidence from Eastern European healthcare systems. This limitation is acknowledged in the Discussion Section.

## 3. Literature Overview

### 3.1. Psychometric Properties and Feasibility

The SAQ demonstrates excellent internal consistency (Cronbach’s α > 0.80), strong test–retest reliability (ICC > 0.75), and responsiveness to clinical change (SRM > 0.40) [11,14,15]. The short version, SAQ-7, maintains validity and prognostic performance while reducing patient burden (correlation with full SAQ r > 0.90) [9,16]. These features make SAQ-7 a practical option for clinical practice, particularly in resource-limited settings.

### 3.2. Prognostic Value

Beyond descriptive symptom assessment, the SAQ gathers robust prognostic information. Lower baseline scores independently predict hospitalizations, myocardial infarction, and death across diverse cohorts [11,14,17]. Major trials and registries—including COURAGE, ISCHEMIA, EuroCTO, PREMIER, TRIUMPH, and CONCORDANCE—consistently demonstrate that the SAQ captures meaningful improvements in angina frequency and quality of life, while also identifying patients at a higher risk of adverse outcomes (Table 1).

Importantly, most of this evidence comes from health systems in North America and Western Europe with broad access to guideline-directed care. In Bulgaria—where late presentation, higher comorbidity, and limited access to specialized interventions are common [5,6,7]—the predictive accuracy of the SAQ requires local validation. Until such data are available, international prognostic associations should be interpreted cautiously.

### 3.3. Alternative PRO Instruments

Several other patient-reported outcome (PRO) tools are available. The SF-36 and EQ-5D are generic instruments with wide validation and cross-disease comparability but limited sensitivity to angina-related change [20,21]. The MacNew questionnaire addresses broader cardiac populations, while the Minnesota Living with Heart Failure Questionnaire (MLHFQ) is specific to HF and of limited relevance for CCSs [22].

In contrast, the SAQ uniquely captures angina frequency and treatment satisfaction in addition to quality of life, explaining its superior responsiveness and prognostic utility [9,14]. While no single PRO instrument is universally optimal, the SAQ provides the most disease-specific insight for CCS populations. Generic tools may still be complementary for health economic evaluations. A comparative summary is presented in Table 2.

Taken together, the evidence confirms that the SAQ is reliable, responsive, and prognostically relevant. The key challenge is therefore not whether the SAQ is valid, but how it should be integrated into structured CCS care pathways. This conceptual framework is outlined in Section 4.

### 3.4. Evidence from Clinical Trials and Registries

The clinical utility of the Seattle Angina Questionnaire (SAQ) has been extensively validated in randomized controlled trials (RCTs) and large-scale registries, where it consistently provided patient-centered insights that complemented traditional endpoints such as mortality or myocardial infarction.

#### 3.4.1. SAQ Use in Major Clinical Trials

The SAQ has played a pivotal role in landmark RCTs. In the COURAGE trial, the addition of PCI to optimal medical therapy improved angina frequency and quality of life, as captured by the SAQ, despite no difference in survival [10]. The ISCHEMIA trial confirmed that patients with frequent angina at baseline experienced the most pronounced improvements in SAQ scores when paired with an invasive strategy, whereas those with minimal symptoms derived little incremental benefit [12]. Similarly, the EuroCTO trial demonstrated that successful recanalization of chronic total occlusions led to significant and sustained improvements in angina frequency, physical limitation, and quality of life compared with optimal medical therapy alone [13]. These findings highlight that while revascularization does not alter long-term survival in stable disease, SAQ scores capture meaningful improvements in symptom burden and health status.

#### 3.4.2. Evidence from Registries

Observational data reinforce the prognostic implications of the SAQ. In the PREMIER registry, lower quality of life scores and dyspnea were independently associated with increased mortality and rehospitalization [17]. The TRIUMPH and CONCORDANCE registries further confirmed that SAQ domains predict functional recovery, readmissions, and long-term outcomes in patients with acute coronary syndromes [18,19]. Importantly, these associations persist after adjusting for traditional risk factors, underscoring the SAQ’s incremental prognostic value.

#### 3.4.3. Summary of Evidence

Table 3 summarizes key trials and registries incorporating the SAQ as a primary or secondary endpoint, illustrating its capacity to detect clinically important benefits, predict prognosis, and capture outcomes most relevant to patients. Clinically, predefined thresholds—such as a 5–8-point improvement in any domain regarded as the minimal clinically important difference (MCID) [14]—enable interpretation and comparability across studies. Emerging evidence also suggests that SAQ-guided management may enhance patient–clinician communication, treatment satisfaction, and adherence, although this remains an evolving research area [23,24].

## 4. A Clinically Oriented Framework: Where the SAQ Fits in CCS Care

### 4.1. Pathway Overview

The integration of the Seattle Angina Questionnaire (SAQ) into clinical care should be framed within the broader management pathway of chronic coronary syndromes (CCSs), as outlined in the 2019 and 2024 ESC Guidelines [3] and the 2023 AHA/ACC Guideline for Chronic Coronary Disease [4]. These guidelines emphasize that optimal care requires not only anatomical and functional assessment but also systematic evaluation of symptoms and quality of life. The SAQ provides a structured way to capture the patient’s perspective, thereby aligning clinical decision-making with outcomes that matter most to patients.

#### 4.1.1. Screening and Baseline Assessment

At the initial presentation of angina or suspected CCS, the SAQ (or its short version, SAQ-7) can serve as a standardized tool for quantifying symptom burden. Unlike routine clinical history, which may underestimate angina frequency, the SAQ provides reproducible, multidimensional insights into physical limitation, angina frequency, and disease-specific quality of life [9,15]. Establishing a baseline score allows physicians to identify patients with significant functional impairment who may benefit most from further evaluation or therapy escalation.

#### 4.1.2. Phenotyping

Following baseline assessment, patients are classified into two major phenotypes: (i) obstructive coronary artery disease (CAD), characterized by epicardial stenoses, and (ii) angina or ischemia without obstructive CAD (ANOCA/INOCA), which is increasingly recognized as a clinically important and prognostically adverse subgroup [3,4]. Within the latter, distinct endotypes such as vasospastic angina (VSA) and microvascular angina (MVA) have been described, each with unique pathophysiological mechanisms and therapeutic implications [25]. SAQ scores can help prioritize patients for further diagnostic work-up, as those with a higher symptom burden may derive greater benefit from phenotype-specific testing.

#### 4.1.3. Diagnostics

For obstructive CAD, the SAQ complements anatomical imaging (CT coronary angiography, invasive angiography) and functional indices such as the fractional flow reserve (FFR) or instantaneous wave-free ratio (iFR). For ANOCA/INOCA, dedicated invasive tests—such as those assessing the coronary flow reserve (CFR), the index of microcirculatory resistance (IMR), and acetylcholine provocation—are increasingly recommended to characterize microvascular dysfunction and vasospastic responses [3,25]. Embedding the SAQ into this step provides a patient-reported benchmark for correlating objective diagnostic findings with subjective health status, enhancing the precision of phenotyping.

#### 4.1.4. Follow-Up and Longitudinal Monitoring

SAQ reassessment is recommended at 4–12 weeks following initiation or adjustment of therapy and again at 6–12 months during stable follow-up. A change of ≥5 points in any domain is generally considered the minimal clinically important difference (MCID) [14]. Persistently low or worsening scores should prompt reconsideration of diagnosis, further testing, or modification of treatment strategy. In this way, the SAQ enables clinicians to move beyond binary outcomes and instead adopt a dynamic, patient-centered model of care.

**Figure 1:**
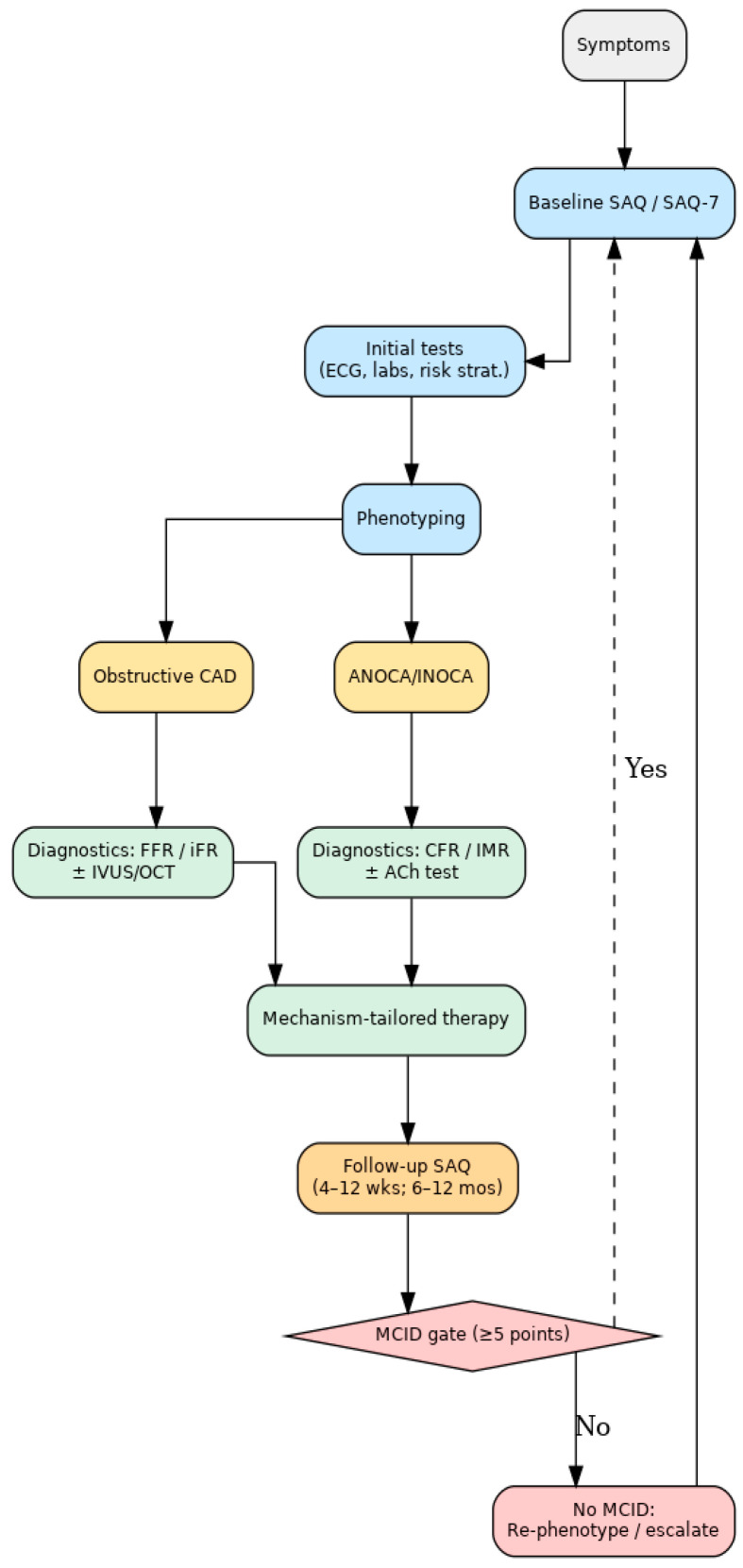
An SAQ-integrated clinical pathway for patients with chronic coronary syndromes. Patients presenting with angina undergo baseline SAQ assessment, initial diagnostic testing, and phenotyping into obstructive vs. ANOCA/INOCA categories. Obstructive CAD is typically assessed with FFR/iFR ± IVUS/OCT, whereas ANOCA/INOCA phenotypes require microvascular or vasospasm testing (CFR, IMR, acetylcholine). Therapy is tailored to the identified mechanism. Follow-up SAQ assessment is performed at predefined timepoints (4–12 weeks, 6–12 months) to evaluate clinically meaningful improvements. An increase of ≥5 points in any SAQ domain represents the minimal clinically important difference (MCID). A lack of improvement triggers re-phenotyping and therapy escalation.

This stepwise pathway illustrates that the SAQ is not a stand-alone questionnaire but rather a decision support tool that links patient-reported outcomes with structured clinical management. By embedding the SAQ at each stage—screening, phenotyping, diagnostics, therapy, and follow-up—clinicians can ensure that CCS care remains both evidence-based and aligned with the lived experience of patients. Figure 1 illustrates the proposed integrated clinical pathway, showing where SAQ assessment complements diagnostic phenotyping and therapy tailoring.

### 4.2. Screening and Triage (Who Should Complete the SAQ and When)

The first practical question in implementing the Seattle Angina Questionnaire (SAQ) is which patients should be screened and at what stage of care. Current European and American guidelines emphasize that symptom assessment and quality of life evaluation should be performed in all patients with suspected or established chronic coronary syndromes (CCSs) [1,3,4]. However, the intensity and frequency of SAQ use should be tailored to the clinical setting.

#### 4.2.1. Initial Presentation

At the time of the first evaluation for suspected angina, the SAQ provides an immediate, standardized measure of symptom burden. Evidence suggests that clinicians often underestimate the frequency and impact of angina when relying solely on history taking [14]. Incorporating the SAQ at this stage ensures that even subtle but clinically meaningful impairments in quality of life are identified, supporting appropriate referral for diagnostic testing.

#### 4.2.2. Patients with Established CCS

In terms of confirmed CAD cases, SAQ screening is particularly valuable for the following patients:

- Patients with ongoing or recurrent angina despite guideline-directed medical therapy (GDMT).
- Patients being considered for revascularization, where baseline health status is crucial for evaluating potential benefit.
- High-risk groups, such as women and patients with multiple comorbidities, in whom angina may present atypically and be under-recognized [25,26].

#### 4.2.3. Post-Intervention or Therapy Initiation

The SAQ should also be administered after major therapeutic decisions—the initiation or intensification of antianginal therapy, PCI, or CABG—to document early changes in symptom burden. This provides a reference point for longitudinal follow-up and helps identify non-responders.

#### 4.2.4. Healthcare System Perspective

From a systems viewpoint, routine SAQ administration may not be feasible in every outpatient encounter, particularly in resource-limited settings such as Bulgaria. A pragmatic approach is to apply the SAQ selectively at key “decision nodes”: baseline evaluation, post-therapy adjustment (4–12 weeks), and long-term follow-up (6–12 months). This strategy balances the need for structured patient-reported outcomes with the realities of limited time and workforce resources [5,6].

In summary, the SAQ should be considered a universal tool for all patients with suspected or established CCS, but its most critical role is in those with moderate-to-severe symptoms, therapy-resistant angina, or pending revascularization. Embedding SAQ administration at these points ensures that clinical decision-making is systematically informed by the patient’s perspective.

### 4.3. Phenotyping: Obstructive CAD Versus ANOCA/INOCA and Endotypes

A key step in the structured management of chronic coronary syndromes (CCSs) is the distinction between patients with obstructive coronary artery disease (CAD) and those with angina or ischemia in the absence of obstructive CAD (ANOCA/INOCA). This classification has major implications for both diagnostic strategies and therapy [3,4]. The SAQ can support this process by systematically identifying patients with substantial symptom burden who are most likely to benefit from more advanced phenotyping.

#### 4.3.1. Obstructive CAD

Patients with epicardial stenoses remain the classical CCS population. In this group, SAQ scores quantify the impact of ischemia on daily life and can guide revascularization decisions. Evidence from COURAGE, ISCHEMIA, and EuroCTO shows that PCI does not consistently improve survival, but it does provide significant symptomatic relief and quality of life gains, particularly in those with severe baseline angina [10,12,13]. Thus, SAQ phenotyping is crucial for identifying patients likely to derive tangible benefit from invasive therapy.

#### 4.3.2. ANOCA/INOCA

Up to 40–50% of patients undergoing coronary angiography for angina symptoms are found to have non-obstructive arteries [3]. These patients often report significant quality of life impairment despite the absence of flow-limiting stenoses. Within this group, further subdivision into endotypes is clinically relevant:

- **Vasospastic angina (VSA):** Characterized by dynamic epicardial spasm, usually responsive to calcium channel blockers and nitrates.
- **Microvascular angina (MVA):** Resulting from impaired microvascular function, best treated with beta-blockers, ACE inhibitors, statins, and selected second-line antianginals.
- **Mixed endotypes:** An overlap of vasospastic and microvascular mechanisms, requiring combination therapy.

Emerging evidence demonstrates that patients with ANOCA/INOCA have higher risks of recurrent angina, impaired quality of life, and adverse long-term outcomes than previously appreciated [25]. For these patients, the SAQ provides an invaluable tool for quantifying symptom severity and monitoring response to targeted therapy.

#### 4.3.3. Clinical Relevance of SAQ Phenotyping

The SAQ does not replace anatomical or functional tests but enriches the phenotyping process as follows:

1. Identifying patients with disproportionate symptom burden relative to anatomical findings.
2. Providing a reproducible baseline for follow-up after targeted diagnostic procedures (e.g., CFR, IMR, acetylcholine testing).
3. Highlighting subgroups (especially women and younger patients) in whom ANOCA/INOCA is more prevalent and often underdiagnosed [25,26].

In summary, the incorporation of the SAQ into CCS phenotyping ensures that patient-reported outcomes are not dissociated from diagnostic decision-making. By differentiating obstructive CAD from ANOCA/INOCA and their respective endotypes, clinicians can better align diagnostic testing and therapy with the mechanisms that truly drive symptoms and quality of life. Table 4 summarizes a practical diagnostic phenotyping matrix linking clinical presentation, imaging, invasive indices, and the likely endotype to suggested next steps in management.

### 4.4. CAD Burden Assessment: Anatomical and Ischemic Evaluation

A critical step in the management of chronic coronary syndromes (CCSs) is the assessment of coronary artery disease (CAD) burden—both anatomical and ischemic. This evaluation guides prognosis, therapeutic decisions, and risk stratification. The Seattle Angina Questionnaire (SAQ) complements this process by quantifying the symptomatic consequences of anatomical disease, ensuring that management decisions reflect not only the extent of stenoses but also their real-life impact on patients.

#### 4.4.1. Anatomical Burden

Coronary computed tomography angiography (CCTA) has become a first-line tool for the non-invasive anatomical assessment of CAD. The CAD-RADS classification system provides a standardized framework for describing coronary lesions, ranging from no stenosis (CAD-RADS 0) to severe multivessel disease (CAD-RADS 5). CAD-RADS has been validated as a predictor of adverse outcomes and is recommended by both ESC and ACC/AHA guidelines [3,4]. However, anatomical severity does not always correlate with symptom burden; patients with diffuse but non-flow-limiting disease may report severe angina, while others with significant stenoses may remain asymptomatic. Here, SAQ scores provide a critical patient-centered complement to CAD-RADS classification.

#### 4.4.2. Ischemia Burden

Functional assessment of ischemia burden—defined as the proportion of the myocardium affected by reversible perfusion defects—has independent prognostic value. Imaging modalities such as stress echocardiography, single-photon emission computed tomography (SPECT), positron emission tomography (PET), and cardiac magnetic resonance (CMR) can quantify ischemia burden. Thresholds of >10% ischemic myocardium have consistently been associated with worse prognosis and may identify patients who could derive greater benefit from revascularization [3]. Yet ischemia burden alone does not capture the patient’s quality of life or symptom experience. Combining SAQ scores with ischemia quantification allows for more nuanced decision-making: a patient with a moderate ischemia burden but profound angina symptoms may still benefit from therapy escalation, whereas a patient with a high ischemia burden but preserved quality of life may be managed conservatively in selected contexts.

#### 4.4.3. Integrated Perspective

The prognostic value of CAD burden is well established, but outcomes such as hospitalization or mortality do not sufficiently capture the full impact of the disease. The SAQ provides an integrated perspective by linking anatomical and ischemic findings with patient-reported outcomes. This integration ensures that treatment decisions are not guided solely by imaging severity but by the actual lived experience of patients, in line with the patient-centered philosophy emphasized by contemporary guidelines [3,4].

In summary, CAD burden assessment defines the anatomical and ischemic substrate of disease, while the SAQ captures its subjective clinical relevance. The combination of these approaches enables a more precise and individualized evaluation of patients with CCS, laying the foundation for mechanism-tailored therapy.

### 4.5. Diagnostic Toolbox: Non-Invasive and Invasive Strategies

Effective management of chronic coronary syndromes (CCSs) requires not only anatomical and ischemic assessment but also precise identification of the underlying pathophysiological mechanism. The diagnostic toolbox therefore spans both non-invasive and invasive modalities, with the Seattle Angina Questionnaire (SAQ) serving as a complementary anchor that links test results with patient-reported outcomes.

#### 4.5.1. Non-Invasive Modalities

- **Exercise treadmill testing (ETT):** Is widely available, but limited sensitivity and specificity; useful in low-to-intermediate risk patients when resources are constrained.
- **Stress echocardiography:** Assesses inducible wall motion abnormalities; offers higher accuracy than ETT and provides additional information on ventricular function.
- **Single-photon emission computed tomography (SPECT) and positron emission tomography (PET):** Quantify perfusion defects and ischemia burden; PET additionally provides absolute myocardial blood flow.
- **Cardiac magnetic resonance (CMR):** Combines perfusion imaging with tissue characterization; useful in younger patients and those with prior infarction or microvascular disease.
- **Coronary CT angiography (CCTA):** Has a high negative predictive value; excellent for anatomical delineation and increasingly integrated into initial evaluation [3,4].

While these tools define ischemia burden and CAD severity, they may not always correlate with the patient’s symptom experience. The SAQ thus provides a structured way to prioritize patients with high symptomatic impairment for further invasive testing or therapeutic escalation.

#### 4.5.2. Invasive Diagnostics in Obstructive CAD

- **Fractional flow reserve (FFR) and instantaneous wave-free ratio (iFR):** Functional indices to determine whether angiographic stenoses are hemodynamically significant.
- **Intravascular imaging (IVUS, OCT):** Provides plaque morphology and lesion characterization, particularly in intermediate stenoses.

SAQ scores help identify patients in whom positive FFR/iFR findings are most clinically relevant—for example, those with frequent angina and low quality of life scores. Conversely, patients with preserved SAQ scores may be managed conservatively despite anatomical lesions.

#### 4.5.3. Invasive Diagnostics in ANOCA/INOCA

The recognition of ANOCA/INOCA as a heterogeneous group has led to increased emphasis on dedicated invasive testing [3,25]:

- **Coronary flow reserve (CFR):** Assesses vasodilator capacity of the coronary microcirculation; abnormal values indicate microvascular dysfunction.
- **Index of microcirculatory resistance (IMR):** Quantifies microvascular resistance independent of epicardial disease.
- **Acetylcholine (ACh) or ergonovine provocation testing:** Identifies epicardial or microvascular vasospasm, confirming a diagnosis of vasospastic angina (VSA).

The SAQ is particularly valuable in these populations, where angiographic findings may appear normal despite significant symptoms. Baseline SAQ scores establish the degree of impairment, while repeat assessments document clinical improvement after mechanism-targeted therapy (e.g., calcium channel blockers for VSA, beta-blockers or ACE inhibitors for MVA).

#### 4.5.4. Complementary Role of SAQ

The diagnostic toolbox provides mechanistic clarity, while the SAQ offers a patient-centered dimension:

- It helps prioritize who undergoes invasive testing.
- It provides a benchmark to interpret whether diagnostic findings translate into meaningful clinical improvement.
- It supports longitudinal monitoring of patients with functional or microvascular disorders, where traditional imaging is less informative.

In summary, the diagnostic toolbox for CCS integrates non-invasive and invasive modalities to characterize anatomical and functional abnormalities. The SAQ complements these tools by ensuring that diagnostic and therapeutic decisions are not made in isolation from the patient’s subjective experience, thereby aligning clinical pathways with both pathophysiology and quality of life outcomes.

### 4.6. Mechanism-Tailored Therapy

Optimal treatment of chronic coronary syndromes (CCSs) depends on aligning therapy with the underlying pathophysiological mechanism. While guideline-directed medical therapy (GDMT) forms the foundation of care for all patients, differentiation between obstructive CAD and ANOCA/INOCA endotypes is essential for targeted and effective management [3,4]. The Seattle Angina Questionnaire (SAQ) provides a systematic way to quantify treatment response across these subgroups, ensuring that therapeutic decisions are not made solely on anatomical grounds but also on the basis of patient-reported health status.

#### 4.6.1. Obstructive CAD

For patients with flow-limiting epicardial stenoses, GDMT—including antiplatelet therapy, statins, ACE inhibitors/ARBs, and beta-blockers—remains the cornerstone. Revascularization with PCI or CABG should be considered for patients with significant ischemia burden or refractory angina. Evidence from COURAGE and ISCHEMIA shows that while revascularization does not reduce long-term mortality compared with medical therapy, it provides substantial improvements in angina frequency and quality of life, particularly in those with severe baseline symptoms [10,12]. SAQ monitoring allows clinicians to identify responders and to document clinically meaningful benefits (≥5-point change in domain scores).

#### 4.6.2. Vasospastic Angina (VSA)

Patients with documented vasospastic angina (positive acetylcholine test or clinical features of variant angina) benefit most from vasodilator therapy. Calcium channel blockers (e.g., amlodipine, diltiazem, verapamil) comprise first-line therapy, often combined with long-acting nitrates [3]. Beta-blockers, particularly non-selective agents, should be avoided as they may worsen spasm. Serial SAQ evaluations are useful for detecting whether symptom relief is achieved and maintained.

#### 4.6.3. Microvascular Angina (MVA)

Patients with microvascular dysfunction (low CFR, high IMR, or microvascular spasm) require a tailored regimen. Beta-blockers, ACE inhibitors, and statins are first-line therapies; second-line agents such as ranolazine or ivabradine may be added in resistant cases [26]. Importantly, SAQ scores in these patients often remain low despite apparently “normal” coronary angiography, underscoring the value of PROMs in this group. Improvements in angina frequency and physical limitation domains provide objective evidence of therapeutic effectiveness beyond conventional imaging.

#### 4.6.4. Mixed Endotypes and Therapy Escalation

A subset of patients present with overlapping mechanisms (e.g., vasospastic plus microvascular dysfunction). These patients often require combination therapy (e.g., CCB + ACE inhibitor + statin) and closer monitoring. SAQ-guided follow-up is particularly valuable in such cases to adjust therapy in a dynamic, patient-centered manner.

#### 4.6.5. Therapy Evaluation and Shared Decision-Making

Beyond pharmacological and revascularization strategies, SAQ-derived information can be integrated into shared decision-making discussions with patients. A demonstrable improvement in SAQ scores reinforces adherence and satisfaction, while persistently low scores prompt re-evaluation of diagnosis, therapy intensification, or referral to specialized centers. This approach aligns clinical care with patient priorities and ensures that therapeutic decisions address not only survival but also health status and daily functioning [14,24,27].

Mechanism-tailored therapy for CCS requires integrating guideline-directed strategies with individualized treatment according to phenotype. The SAQ provides a reproducible and sensitive measure of treatment response across all subgroups—obstructive CAD, vasospastic angina, microvascular angina, and mixed endotypes—thus bridging the gap between anatomical findings and the patient’s lived experience. Table 5 provides a simplified therapy map by endotype, including primary and adjunctive treatment strategies as well as the expected timeframe for SAQ response.

### 4.7. Where the SAQ Plugs in and How It Informs Decisions

The practical value of the Seattle Angina Questionnaire (SAQ) lies in its systematic integration into key stages of patient care. Rather than functioning as a stand-alone survey, the SAQ should be embedded at predefined timepoints to guide therapy decisions, support follow-up, and trigger escalation of care when necessary.

#### 4.7.1. Baseline Assessment

At the time of the first evaluation or diagnosis of chronic coronary syndrome (CCS), the SAQ provides a standardized measure of symptom burden and quality of life. Establishing a baseline score enables clinicians to quantify disease impact beyond anatomical findings. This initial assessment is particularly important for stratifying patients with moderate-to-severe symptoms who may require invasive testing or consideration of revascularization [3,4].

#### 4.7.2. Post-Therapy or Post-Procedure Follow-Up

The SAQ should be repeated after the initiation or intensification of anti-anginal therapy (typically at 4–12 weeks) and after revascularization procedures (PCI or CABG). A change of ≥5 points in any SAQ domain is considered the minimal clinically important difference (MCID), while larger changes (≥10 points) reflect robust clinical improvement [14]. Documenting these changes ensures that the effectiveness of treatment is judged from the patient’s perspective and not solely from imaging or laboratory parameters.

#### 4.7.3. Longitudinal Monitoring

During stable follow-up (6–12 months), the SAQ can track symptom trajectories. Patients with persistently low or deteriorating scores despite optimal medical therapy should be flagged for further diagnostic work-up or reconsideration of therapeutic strategy. Conversely, patients with stable or improved scores may be managed conservatively with confidence, reducing unnecessary testing.

#### 4.7.4. Decision Triggers

SAQ scores provide actionable thresholds:

- **Angina Frequency < 60** or **Quality of Life < 50** → high symptom burden; consider escalation of therapy or invasive evaluation.
- **Minimal change after GDMT initiation** → consider phenotyping for ANOCA/INOCA or referral for revascularization.
- **Decline in Physical Limitation despite therapy** → reassess for comorbidities (e.g., HFpEF, COPD) that may contribute to reduced functional capacity.

#### 4.7.5. Shared Decision-Making

By presenting SAQ results in a structured, visual format, clinicians can engage patients in shared decision-making. Patients can gain clarity on how their symptoms compare over time and how different therapies have affected their quality of life, which supports adherence and trust in the therapeutic plan [24,27].

#### 4.7.6. Summary

The SAQ informs clinical decision-making at three key points—baseline, post-therapy/procedure, and longitudinal follow-up. With predefined thresholds such as the MCID, the SAQ provides objective triggers for escalation or modification of therapy, ensuring that treatment remains closely aligned with the patient’s experience.

### 4.8. Implementation Metrics and Registry Fields

For the Seattle Angina Questionnaire (SAQ) to be implemented successfully in routine practice, it must be incorporated not only as a clinical tool but also as part of a broader quality measurement and registry framework. This requires defining a minimum dataset, establishing meaningful quality indicators, and ensuring interoperability across centers and health systems.

The minimum dataset should include domain-level SAQ results, ideally covering Physical Limitation, Angina Frequency, and Quality of Life, with the option to capture the full five domains when feasible. In settings where time is limited, the SAQ-7 can serve as a concise and validated alternative, providing a global score that preserves prognostic value. To support evaluation and comparability, registries should also capture completion rates (the proportion of eligible patients who complete the questionnaire) and the timing of administration (e.g., baseline, short-term follow-up at 4–12 weeks, and long-term follow-up at 12 months). Such standardization ensures that patient-reported outcomes are collected consistently and that they can be meaningfully interpreted across institutions. In addition to aggregated indicators, a minimum dataset should be collected at each patient encounter to ensure standardization and enable longitudinal comparisons across centers. This dataset should include baseline and follow-up SAQ assessments, cardiovascular risk profile, diagnostic and therapeutic status, and phenotype classification. To facilitate clinical use, Box 1 outlines a practical minimum dataset for an SAQ-driven visit. Such a structured template can be incorporated into electronic health records as a smart phrase or checklist, ensuring the completeness of documentation and facilitating integration into national registries.

Box 1 **Minimum dataset for SAQ-driven visit.**

☐ Baseline SAQ (full or SAQ-7)

☐ Cardiovascular risk profile (hypertension, diabetes, dyslipidemia, smoking)

☐ Medication history (anti-anginals, secondary prevention)

☐ Diagnostic work-up status (non-invasive, invasive)

☐ Phenotype label (Obstructive/MVA/VSA/Mixed)

☐ SAQ follow-up timepoint (weeks)

☐ MCID achieved? (Yes/No; which domain)

☐ Next step (therapy intensification, re-phenotyping, referral to interventional testing)

From a quality of care perspective, SAQ-based metrics offer unique opportunities to establish patient-centered performance indicators. These could include, for example, the percentage of patients with CCS who have an SAQ documented at baseline evaluation, the proportion with repeat assessment within one year, and the percentage achieving a minimal clinically important difference (MCID) of at least five points in Angina Frequency or Quality of Life domains after therapy or revascularization. Another important indicator is the prevalence of persistent angina despite guideline-directed medical therapy, as this identifies a subgroup at high risk of impaired outcomes and guides system-level resource allocation. Unlike traditional performance measures focused on procedural success or mortality, these indicators reflect the outcomes that matter most to patients.

Experience from international registries has shown that integrating patient-reported outcomes (PROs) into structured datasets facilitates benchmarking between centers, drives local quality improvement, and informs policy decisions [8,23,27]. For Bulgaria, where cardiovascular registries remain underdeveloped, the integration of SAQ into future national or regional databases could represent a step forward in aligning with European initiatives such as the ESC’s EuroHeart program. In addition to improving clinical care, such integration would expand the country’s capacity to contribute to international collaborative research and policy development.

In summary, embedding the SAQ into registries through a clearly defined minimum dataset and patient-centered quality indicators transforms it from a descriptive research tool into a mechanism for accountability and system-wide improvement. By capturing not only survival and procedural success but also health status and quality of life, registries can provide a more comprehensive picture of care outcomes and guide the evolution of patient-centered cardiology in Bulgaria.

### 4.9. Workflow and Digital Integration in Bulgaria

Successful implementation of the Seattle Angina Questionnaire (SAQ) in Bulgaria will depend on adapting it to the realities of local healthcare workflows and digital infrastructure. Beyond translation and validation, a major challenge is ensuring that SAQ administration is feasible in busy clinical environments and that results are seamlessly integrated into electronic health records (EHRs) and national registries.

#### 4.9.1. Clinical Workflow Integration

In outpatient cardiology settings, consultations are typically brief and constrained by workforce shortages. To minimize additional burden, the SAQ should be administered in the waiting area or via digital platforms prior to the clinical encounter. This approach ensures that results are available to the physician in real time without extending consultation length. In inpatient settings, administration could be embedded into admission and discharge protocols, providing structured data at critical care transitions. Importantly, clinicians need training on how to interpret SAQ scores and how to communicate them effectively to patients, transforming raw numbers into actionable insights that support shared decision-making.

#### 4.9.2. Digital Tools and Interoperability

The fragmented nature of Bulgaria’s health information systems presents another barrier. Hospitals and outpatient practices use heterogeneous EHR platforms with limited interoperability, hindering the aggregation of standardized patient-reported outcome (PRO) data. A feasible initial step would be the development of a simple, web-based or app-based SAQ platform capable of automatic scoring and data export in standardized formats (e.g., HL7 FHIR). This would allow data to be uploaded into local hospital systems and, in the long term, into national cardiovascular registries. Visual dashboards that summarize domain scores and highlight changes over time could further support clinical interpretation and improve patient engagement.

#### 4.9.3. Pilot Implementation and Scalability

Given these challenges, a stepwise approach is essential. Pilot projects in high-volume academic and tertiary cardiology centers should test the feasibility of electronic SAQ administration, assess completion rates, and evaluate clinician and patient acceptability. Data from such pilots could then inform broader rollout, including integration into national registries or linkage with the planned digital health initiatives in Bulgaria. Scalability will also depend on alignment with reimbursement mechanisms and on demonstrating that SAQ implementation improves efficiency—by reducing redundant diagnostic testing, supporting more appropriate use of PCI, and facilitating long-term monitoring of treatment effectiveness.

Embedding the SAQ into Bulgarian cardiology requires workflow redesign, digital solutions, and systemic support. By leveraging electronic platforms, aligning with registry development, and starting with pilot projects in academic centers, Bulgaria can overcome infrastructural barriers and establish a foundation for patient-centered care. Ultimately, digital integration of the SAQ has the potential to transform how angina and quality of life are measured, documented, and acted upon in routine practice.

## 5. Implementation of the Seattle Angina Questionnaire in Bulgaria

### 5.1. Epidemiological and Clinical Context

Bulgaria continues to face an exceptionally high burden of cardiovascular disease, which remains the leading cause of morbidity and mortality nationwide. According to the European Society of Cardiology Atlas of Cardiology, the country consistently ranks among the European states with the highest age-standardized mortality from ischemic heart disease, and cardiovascular causes account for more than 60% of all annual deaths [5,7]. Despite significant progress in acute care and wider access to percutaneous coronary interventions, these figures have remained alarmingly stable over the past few decades. Such persistence underscores not only structural weaknesses in the healthcare system but also the absence of systematic patient-centered strategies for managing chronic disease. The prevalence of angina and ischemic heart disease is particularly pronounced, driven both by demographic factors such as rapid population aging and by the high prevalence of modifiable risk factors including hypertension, diabetes, dyslipidemia, and tobacco use [1,27].

#### 5.1.1. Structural and Contextual Factors Influencing Feasibility

The feasibility of implementing the SAQ in Bulgaria must be considered within the broader constraints of the national healthcare system. Electronic health record (EHR) infrastructure remains fragmented, with heterogeneous software platforms and limited interoperability between hospitals and outpatient practices, creating barriers to systematic data capture and analysis [6]. Outpatient visits are often brief and constrained by workforce shortages [7], leaving limited time for the administration and interpretation of PRO instruments. Furthermore, the absence of local cardiovascular registries incorporating patient-reported outcomes reduces opportunities for benchmarking and longitudinal follow-up [5,8]. Financial and organizational barriers—including underfunding of preventive care, limited reimbursement mechanisms for PRO implementation, and high regional variability in healthcare access—further complicate widespread adoption [6].

These systemic constraints are not only organizational but also directly limit the capacity to generate actionable information from SAQ assessments. Fragmented electronic systems hinder standardized collection and longitudinal storage of PRO data, while the lack of national registries precludes pooled analysis or benchmarking across centers. Workforce shortages and time-limited consultations reduce opportunities for meaningful interpretation and discussion of SAQ scores during patient visits. Furthermore, in the absence of formal reimbursement or guideline-based integration, SAQ data risk being collected but underutilized in actual clinical decision-making. Together, these factors highlight that implementation must address not only the translation and validation of the tool, but also the infrastructural and organizational prerequisites for its effective use [6,7].

#### 5.1.2. Cultural and Linguistic Adaptation

A critical prerequisite for the implementation of the SAQ in Bulgaria is the assurance of not only accurate linguistic translation but also conceptual and cultural equivalence. Patient-reported outcome instruments are highly sensitive to language and context, as even subtle semantic differences may alter the interpretation of items and undermine comparability across populations. The SAQ domains of Treatment Satisfaction and Disease-specific Quality of Life are particularly vulnerable to cultural variation. In the Bulgarian healthcare environment, perceptions of treatment satisfaction may be influenced by factors such as waiting times, access to diagnostic procedures or medications, and communication style between patient and physician, rather than by the quality of the treatment itself. Similarly, assessments of disease-specific quality of life may be confounded by broader socioeconomic determinants, including income, employment stability, or cultural attitudes towards health and aging, which may differ substantially from Western populations where the SAQ was initially developed and validated.

To address these challenges, cultural adaptation must follow internationally established frameworks [28,29]. These recommend a rigorous multi-step process:

1. Forward translation of the SAQ into Bulgarian by at least two independent bilingual translators with expertise in medical terminology.
2. Synthesis and reconciliation of the translations by an expert panel including cardiologists, methodologists, and linguists to ensure semantic and conceptual accuracy.
3. Backward translation into English by translators blinded to the original version to confirm the fidelity of meaning.
4. Expert committee review to resolve discrepancies and ensure that the adapted version is both linguistically accurate and culturally appropriate.
5. Cognitive debriefing interviews with a representative sample of Bulgarian patients with chronic coronary syndrome to test the clarity, relevance, and acceptability of each item. Particular attention should be paid to domains that may have culturally specific interpretations, such as Treatment Satisfaction and Quality of Life.
6. Pilot testing of the provisional Bulgarian SAQ in clinical settings, followed by comprehensive psychometric validation (including reliability, internal consistency, test–retest reproducibility, construct validity, and responsiveness to clinical change).

This process may also reveal the need for minor contextual modifications, for example clarifying examples used in the Physical Limitation domain to reflect activities common in Bulgaria, or adapting wording in the Treatment Satisfaction domain to align with patient expectations of outpatient care delivery. Importantly, such modifications must preserve the underlying construct of the original instrument to ensure cross-cultural comparability.

Without these steps, there is a risk that the Bulgarian version of the SAQ would fail to capture the intended constructs, leading to biased or uninterpretable results. In contrast, rigorous cultural adaptation would provide a validated, context-sensitive instrument that maintains equivalence with the original SAQ while ensuring relevance and accuracy for Bulgarian patients. Similar methodological challenges have been reported in other Eastern European countries, where cultural adaptation of health-related quality of life instruments has been conducted in Poland [30] and Iran [31]. These experiences highlight that even in culturally proximate populations, rigorous validation is essential to ensure conceptual equivalence and reliability. This process is therefore not a methodological formality but an essential foundation for the successful integration of patient-reported outcomes into cardiovascular care and research in Bulgaria.

#### 5.1.3. Systemic Determinants of Cardiovascular Outcomes in Bulgaria

Bulgaria continues to report among the highest rates of cardiovascular morbidity and mortality in Europe, a pattern that has persisted despite gradual improvements in diagnostic and therapeutic capacity. These outcomes are not attributable solely to the biological burden of disease but are strongly shaped by systemic deficiencies in the national healthcare system.

The first structural determinant is the limited access to specialized cardiovascular care. Specialist services are unevenly distributed, with a higher concentration in large urban centers and relative scarcity in rural areas. Patients in remote regions often face substantial geographic and financial barriers to accessing timely diagnostics, invasive procedures, and secondary prevention programs [5,6,7].

The second determinant is the workforce shortage in cardiology and primary care. Bulgaria has one of the lowest densities of practicing cardiologists and general practitioners per capita in the European Union, a problem exacerbated by the migration of healthcare professionals and aging of the current workforce [6,7]. This shortage contributes to long waiting times, shortened outpatient consultations, and limited opportunities for structured follow-up and patient education. These constraints directly undermine the feasibility of systematic patient-reported outcome collection and interpretation in routine practice.

The third determinant is the chronic underfunding of primary prevention and rehabilitation programs. While hospital-based interventions are relatively well developed, investment in community-level prevention, risk factor modification, and cardiac rehabilitation remains insufficient [5,6]. As a result, patients frequently present with advanced disease, recurrent events, and complex comorbidity profiles, further compounding the challenges of improving survival and quality of life.

These systemic limitations explain much of the discrepancy between Bulgaria and Western European countries in cardiovascular outcomes. They also clarify that the introduction of patient-reported outcomes such as the SAQ cannot be expected to resolve these deep-rooted structural issues. Instead, the SAQ should be regarded as a complementary tool, capable of capturing patient-centered information on symptom burden and quality of life. Its role is not to substitute for systemic reforms but to add value by enabling more nuanced clinical decision-making, identifying subgroups of patients at risk of impaired outcomes, and contributing data for benchmarking and quality improvement initiatives.

Importantly, any attempt to implement the SAQ in Bulgaria must be carefully aligned with existing healthcare priorities and resource constraints. Integration should be incremental and context-sensitive, beginning with pilot programs in high-volume tertiary centers where infrastructure and expertise are available and subsequently expanding to broader networks once digital platforms, workforce capacity, and reimbursement mechanisms are in place. In this way, the SAQ could serve as a catalyst for advancing patient-centered care, but its success will ultimately depend on parallel investments in systemic reforms targeting access, workforce development, and preventive care.

Alignment with international frameworks does not in itself ensure better outcomes; in resource-constrained settings, effects must be demonstrated through pilot implementation, monitoring (e.g., uptake/completion rates), and outcome evaluation (e.g., changes in SAQ scores, utilization, readmissions) before broader scale-up is considered.

#### 5.1.4. Limitations of SAQ Implementation in Bulgaria

While the Seattle Angina Questionnaire provides valuable disease-specific insights into symptom burden and quality of life, its implementation in Bulgaria must be carefully evaluated in light of several limitations.

First, as with all patient-reported outcome measures, SAQ results are vulnerable to measurement bias. Recall bias may occur when patients inaccurately report the frequency or severity of symptoms over the recall period. Response bias can distort outcomes if patients systematically over- or underestimate their health status, while social desirability bias may influence individuals to provide answers they perceive as more acceptable to physicians rather than fully reflecting their lived experience. These risks may be particularly relevant in Bulgaria, where traditional physician–patient dynamics and cultural norms may discourage patients from openly reporting dissatisfaction or limitations [16,28,29]. Mitigation strategies include standardized administration (e.g., self-completed rather than interviewer-led questionnaires), clear communication regarding confidentiality, and electronic data capture platforms to reduce social pressure. While these biases cannot be entirely eliminated, acknowledging them is essential for correct interpretation and integration of SAQ results.

Second, until rigorous translation and cultural validation are completed, there remains a risk of conceptual misinterpretation of key domains, particularly Treatment Satisfaction and Disease-specific Quality of Life. These constructs may be interpreted differently in the Bulgarian context, and without proper validation, this may undermine the reliability and comparability of scores [28,29].

Third, practical feasibility must be considered. Workforce shortages and brief outpatient consultations limit the time available for administering and interpreting PRO instruments. This may reduce the likelihood of consistent uptake in daily practice [6,7].

Fourth, the lack of digital integration into national electronic health record systems and cardiovascular registries remains a major barrier. Without interoperable platforms, SAQ data cannot be aggregated, benchmarked, or tracked longitudinally across centers [5,8].

Finally, there is currently no evidence on cost-effectiveness or return on investment for SAQ implementation in Bulgaria. This gap is critical for policymakers, as decisions to adopt PRO instruments must be weighed against other pressing healthcare needs within a resource-constrained system [6].

Taken together, these limitations highlight the importance of viewing the SAQ not as a universal solution but as a promising complementary tool. Its successful adoption in Bulgaria will depend on rigorous cultural validation, pilot testing in high-volume centers, the development of digital infrastructure, and integration within broader systemic reforms aimed at strengthening cardiovascular care.

#### 5.1.5. Integration into Patient-Centered Decision-Making

Although the SAQ has the potential to support shared decision-making, its effectiveness in this role is not automatic. Incorporating SAQ results into clinical consultations requires clinicians to be trained not only in interpreting domain scores but also in communicating them meaningfully to patients. Time constraints in outpatient practice, already limited by workforce shortages, pose an additional barrier to the sustained use of PRO data during consultations [6,7]. Moreover, cultural factors may influence how Bulgarian patients respond to receiving structured, quantitative feedback about their symptoms and quality of life, with some patients valuing the feedback while others may perceive it as abstract or impersonal. These challenges underscore that collecting PRO data is not equivalent to achieving patient-centered care. Instead, successful integration requires workflow redesign, educational initiatives, and digital tools that allow for rapid scoring, visualization, and interpretation of SAQ results within routine practice.

#### 5.1.6. Economic Considerations

The economic feasibility of implementing the SAQ in Bulgaria represents a critical dimension that requires careful evaluation. At present, no cost-effectiveness analyses or return-on-investment studies have been conducted to assess the integration of SAQ into the Bulgarian healthcare system. This gap is particularly important, as decisions on introducing new patient-reported outcome (PRO) instruments must take into account opportunity costs and the prioritization of limited healthcare resources.

International evidence suggests that routine use of PRO measures can lead to improved efficiency of care. Studies in other clinical contexts have shown that systematic symptom monitoring with PROs may reduce avoidable hospitalizations, improve adherence to therapy, and contribute to better overall outcomes, including gains in quality-adjusted life years (QALYs) [24,32]. While such benefits are plausible for the SAQ in chronic coronary syndrome, their realization in Bulgaria remains to be empirically demonstrated.

The costs of national implementation would include the translation and validation of the instrument, the training of healthcare professionals, the time and resources needed for questionnaire administration, and the development of electronic infrastructure for data capture, scoring, and integration into existing health information systems. These direct expenditures must be weighed against potential benefits, including earlier identification of patients with a high symptom burden, better targeting of therapeutic interventions, reduced duplication of diagnostic procedures, and improved patient engagement in shared decision-making.

Given the absence of local evidence, future pilot projects for SAQ implementation in Bulgaria should incorporate a dedicated health economic evaluation component. Such analyses should account for both direct and indirect costs, assess long-term patient outcomes, and model system-level effects on resource utilization. Only through this approach can the return on investment of SAQ adoption be adequately established, thereby providing a sound basis for policy decisions in a resource-constrained healthcare environment.

### 5.2. The Rationale for Implementing the SAQ

In this epidemiological context, the introduction of validated patient-reported outcome measures is not a methodological luxury but rather a pressing necessity. The Seattle Angina Questionnaire (SAQ), by providing a reproducible and multidimensional assessment of symptom burden and quality of life, has the potential to transform the evaluation of patients with chronic coronary syndrome in Bulgaria. In a healthcare environment that continues to rely heavily on mortality and hospitalization data, the SAQ offers a complementary perspective that captures the lived experience of patients. Its integration into clinical practice would allow for the systematic documentation of treatment benefits that are otherwise difficult to quantify, thereby aligning therapeutic decision-making with outcomes that matter most to patients [6,9,15]. Furthermore, the prognostic capacity of the SAQ—demonstrated through its ability to predict hospitalizations and mortality independently of traditional risk factors—underscores its utility as a dual-purpose instrument for both clinical care and risk stratification [11,32].

### 5.3. Potential Benefits of SAQ Integration

The potential benefits of adopting the SAQ extend across multiple levels of the healthcare system. At the individual patient level, routine application of the SAQ would enhance the accuracy of symptom assessment, allowing for timely adjustments of medical therapy or revascularization strategies based on patient-reported data rather than solely based on physician judgment. At the institutional level, the aggregation of SAQ results across hospitals could serve as a valuable tool for monitoring performance, enabling comparisons of outcomes and quality indicators between centers. This benchmarking process, already introduced in other European healthcare systems, has been shown to foster accountability and stimulate local quality improvement initiatives [8]. At the national level, systematic collection of SAQ data could generate a new dimension of health metrics, complementing conventional indicators such as mortality or readmission rates. Such information could inform health policy, guide resource allocation, and provide a more comprehensive understanding of the societal burden of ischemic heart disease. Moreover, by aligning Bulgaria with international standards that increasingly require PROs in multicenter registries and clinical trials, SAQ integration would strengthen the country’s research capacity and open opportunities for international collaboration.

### 5.4. Anticipated Barriers and Challenges

Despite these advantages, the implementation of the SAQ in Bulgaria is likely to face several challenges. The foremost among them is the need for rigorous linguistic translation and cultural adaptation, a process that must ensure both semantic and conceptual equivalence with the original instrument. Equally important is the establishment of the psychometric validity of the Bulgarian version, which requires testing in diverse and representative patient cohorts. Infrastructure also presents a significant obstacle, as electronic health record systems remain fragmented and unevenly distributed across institutions. Integrating PROs such as the SAQ into these digital platforms will require targeted investments in technology and interoperability. Beyond technical considerations, the adaptation of clinical workflows poses another hurdle, as clinicians and nursing staff will need adequate training to administer the questionnaire and to interpret its results meaningfully. Finally, long-term sustainability will depend on the political and financial commitment to embed PROs within the framework of national cardiovascular programs, supported by appropriate reimbursement strategies and performance-based incentives.

### 5.5. Roadmap for Implementation

**Figure 2:**
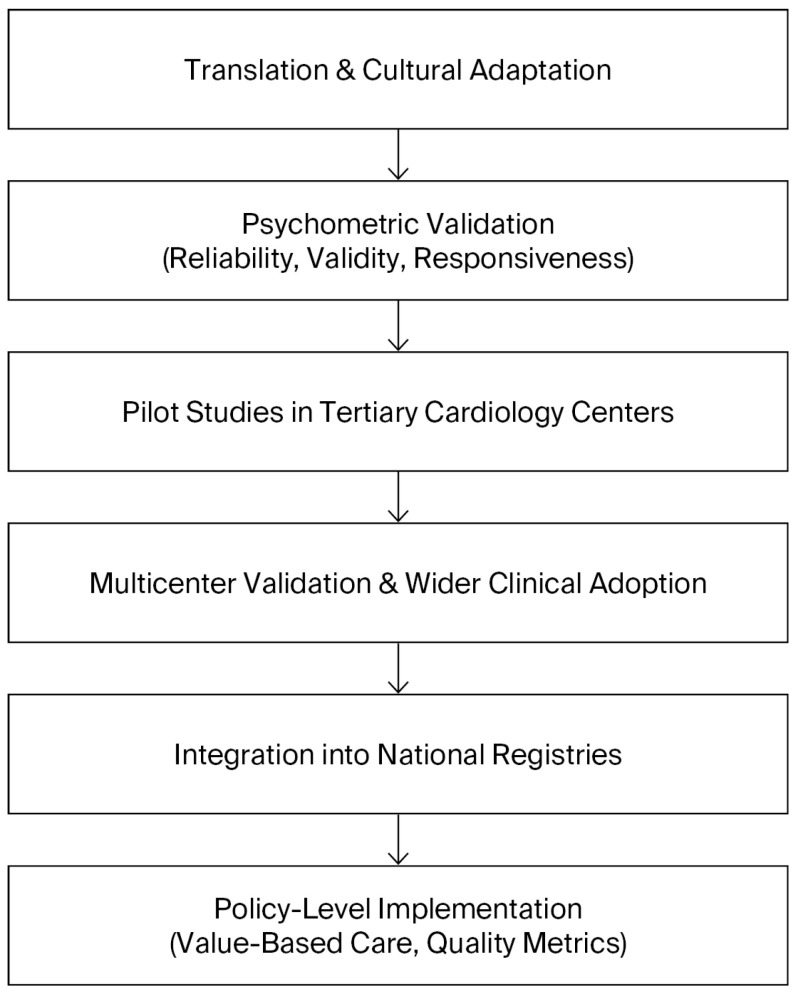
A roadmap for implementing the Seattle Angina Questionnaire (SAQ) in Bulgaria.

A roadmap for the implementation of the SAQ in Bulgaria should therefore be envisioned as a staged process that balances feasibility with ambition. The initial phase must focus on translation, cultural adaptation, and validation of the instrument, ensuring its reliability and acceptability among Bulgarian patients. Once validated, pilot projects in tertiary cardiology centers should assess the feasibility of routine SAQ use in clinical practice, including its integration into existing workflows and information systems. Insights from these pilots will be crucial for refining the approach before broader implementation. The subsequent phase should aim to achieve systematic integration into electronic health records, with automated scoring and reporting to facilitate clinical use and minimize administrative burden. Ultimately, the aim should be to achieve national adoption of the SAQ as a standardized measure of care quality, formally recognized in clinical guidelines and national cardiovascular registries. This progressive trajectory can be conceptualized as a stepwise roadmap, beginning with translation and validation, followed by pilot implementation, integration into clinical workflows, and culminating in national adoption. The stages of this roadmap are summarized in Figure 2, which illustrates the sequential strategy for implementing the SAQ in Bulgaria. In this way, the introduction of the SAQ could serve as a catalyst for a more patient-centered, evidence-based, and transparent cardiovascular care system, aligned with contemporary international practice.

## 6. Discussion

### 6.1. The SAQ as an Evidence-Based Patient-Reported Outcome

The Seattle Angina Questionnaire (SAQ) has been validated across multiple clinical settings, including randomized trials and real-world registries, and consistently demonstrates its ability to capture health status outcomes beyond conventional measures such as mortality and myocardial infarction. Improvements in angina frequency, physical limitation, and quality of life—detected by the SAQ—often occur in the absence of differences in survival, highlighting the instrument’s unique capacity to reflect outcomes most meaningful to patients. These findings, summarized in our literature overview, provide the evidentiary foundation for considering the SAQ as more than a research tool: it is a clinically relevant instrument with both prognostic and therapeutic implications.

### 6.2. Integration into a Structured CCS Pathway

Building on this evidence, the present review proposes a structured framework for the integration of the SAQ into the management of patients with chronic coronary syndromes (CCSs). In contrast to its purely descriptive use, the SAQ should be implemented at clearly defined junctures of the clinical pathway. At the screening stage, it can support triage by identifying patients with a high burden of angina symptoms who may benefit from more intensive diagnostic evaluation. During phenotyping, SAQ data can complement anatomical and physiological findings by documenting the lived impact of obstructive coronary artery disease as well as angina without obstructive coronary arteries (ANOCA/INOCA). In the diagnostic phase, the SAQ can offer an anchor for interpreting results from advanced tools such as coronary CT angiography, invasive physiology, or vasoreactivity testing. Therapy can then be tailored not only to pathophysiological endotypes but also to symptom burden and quality of life impairments, ensuring treatment strategies that are both evidence-based and patient-centered. Finally, in the follow-up stage, repeated SAQ assessments can allow for longitudinal monitoring of disease trajectory, early identification of patients with persistent angina despite guideline-directed medical therapy, and timely adjustment of interventions. This structured approach situates the SAQ within a broader continuum of care, transforming it from a descriptive questionnaire into a decision support tool.

### 6.3. Barriers and Enablers in the Bulgarian Context

Translating this conceptual framework into practice requires attention to national healthcare realities. In Bulgaria, several systemic barriers have historically limited the use of patient-reported outcomes (PROs). Electronic health records remain fragmented, with limited interoperability between hospitals and outpatient settings, hindering standardized data collection and longitudinal follow-up. Outpatient consultations are typically brief and constrained by workforce shortages, reducing the time available for a structured assessment of health status. Moreover, national registries capturing PRO data are lacking, and reimbursement mechanisms currently offer no incentives for systematic use of instruments such as the SAQ. Cultural and linguistic adaptation is another critical prerequisite, since domains such as Treatment Satisfaction and Disease-specific Quality of Life may be interpreted differently by Bulgarian patients compared with populations where the instrument was originally validated. Evidence from Poland [31] and Iran [33] illustrates that successful adaptation of the SAQ and related tools is feasible in Eastern European contexts, providing a useful precedent for Bulgarian implementation efforts. These obstacles highlight that implementation requires not only psychometric validation but also infrastructural and policy-level support.

At the same time, there are enablers that create opportunities for progress. Bulgaria is currently expanding digital health initiatives at the national level, including electronic prescription systems and nascent plans for interoperable registries. High-volume academic centers already demonstrate capacity for piloting innovations, particularly in interventional cardiology where experience with PROMs is growing. Moreover, alignment with European Society of Cardiology (ESC) recommendations and ongoing collaboration within the EuroHeart project provide opportunities to harmonize data collection and benchmarking across Europe.

### 6.4. Educational and Clinical Implications

Integrating the SAQ into clinical workflows offers several practical benefits. First, it enhances shared decision-making by making symptom burden and quality of life impairments explicit and measurable. This helps align therapeutic choices with patient priorities, thereby improving treatment satisfaction and adherence. Second, the SAQ provides a reproducible metric for evaluating therapy effectiveness, allowing clinicians to monitor progress in a standardized way. Third, aggregated SAQ data can serve as quality indicators at the institutional and national level, complementing conventional performance measures such as PCI success rates or readmission frequencies. Introducing the SAQ into clinical registries could therefore improve accountability, stimulate local quality improvement initiatives, and strengthen Bulgaria’s capacity for international collaboration. To realize these benefits, however, clinicians require training not only in administering the questionnaire but also in interpreting domain scores, integrating them into consultations, and communicating results effectively to patients. Educational programs for cardiology trainees and continuing medical education modules for practicing physicians should be considered as part of implementation strategies.

### 6.5. Future Directions

Future efforts should prioritize a staged approach to implementation. The first step is rigorous translation, cultural adaptation, and psychometric validation of the Bulgarian SAQ, following internationally recognized methodologies. Pilot projects in academic centers should then evaluate feasibility, completion rates, and user acceptability, while integrating electronic solutions for data capture and visualization. These pilots should also incorporate economic evaluation to assess cost-effectiveness and sustainability. Once feasibility is demonstrated, expansion into broader registries and health information systems can be pursued, ideally linked to the ongoing digital health reforms in Bulgaria. Ultimately, national adoption will depend on endorsement by professional societies, integration into clinical guidelines, and alignment with reimbursement models. Through such a structured process, the SAQ can evolve from a research instrument into a cornerstone of patient-centered cardiology in Bulgaria.

In summary, the SAQ is a validated, prognostically meaningful instrument that captures dimensions of chronic coronary syndromes not reflected by traditional clinical endpoints. Its integration into a structured pathway—encompassing screening, phenotyping, diagnostics, tailored therapy, and longitudinal follow-up—offers clear clinical and educational value. While systemic barriers in Bulgaria remain considerable, the combination of cultural adaptation, pilot implementation, digital integration, and registry development provides a feasible roadmap for progress. With coordinated efforts between clinicians, academic institutions, and policymakers, the SAQ has the potential to align Bulgarian cardiology with international best practices and to ensure that care addresses outcomes that matter most to patients.

## 7. Conclusions

The Seattle Angina Questionnaire (SAQ) is one of the most validated disease-specific patient-reported outcome measures in cardiology, with robust psychometric performance and consistent prognostic value across trials and registries. Beyond its descriptive role, the SAQ provides unique insights into symptom burden and quality of life that are not captured by conventional endpoints such as survival or hospitalization.

This review highlights how the SAQ can be integrated into a structured clinical pathway for chronic coronary syndromes (CCSs), spanning from initial screening and phenotyping, through advanced diagnostics and mechanism-tailored therapy, to longitudinal follow-up. Such an approach transforms the SAQ from a research instrument into a practical decision support tool, aligning therapeutic strategies with patient-reported outcomes and facilitating shared decision-making.

In Bulgaria, the introduction of the SAQ requires a staged and context-sensitive process. Translation and cultural adaptation must be followed by psychometric validation, pilot testing in academic and high-volume centers, and gradual integration into electronic health records and national registries. Embedding SAQ data into minimum datasets and quality indicators will enhance benchmarking, support accountability, and strengthen research capacity. Ultimately, national adoption will depend on alignment with professional society endorsement, digital health reforms, and sustainable reimbursement models.

In conclusion, the SAQ represents an opportunity to advance patient-centered cardiology in Bulgaria, harmonizing local practice with international standards. By systematically capturing what matters most to patients—symptoms, functional status, and quality of life—the SAQ can complement existing outcome measures and support a more comprehensive, equitable, and value-based approach to cardiovascular care.

## Figures and Tables

**Table 1:** The prognostic value of the Seattle Angina Questionnaire (SAQ) in major trials and registries.

Study/Registry	Population	Key SAQ Domains	Main Prognostic Finding
Spertus et al., 2002 [11]	Outpatients with CAD (USA)	Angina Frequency, QoL	Lower baseline scores independently predicted mortality and hospitalization
PREMIER [17]	Post-MI survivors (USA)	QoL, Angina Frequency, Dyspnea	Poorer scores were associated with higher 1-year mortality and rehospitalization
COURAGE [10]	Stable CAD; PCI + OMT vs. OMT	Angina Frequency, QoL	PCI improved SAQ scores despite no survival benefit
ISCHEMIA [12]	Moderate–severe ischemia	Angina Frequency, QoL	Invasive strategies improved SAQ scores, especially in symptomatic patients
EuroCTO [13]	CTO patients; PCI vs. OMT	Angina Frequency, Physical Limitation, QoL	Successful PCI provided sustained symptomatic and QoL benefit
TRIUMPH/CONCORDANCE [18,19]	ACS survivors (USA, Australia)	QoL, Physical Limitation	Lower SAQ scores predicted readmissions and impaired recovery

**Table 2:** Comparison of selected PRO instruments relevant to chronic coronary syndrome.

Instrument	Type	Main Domains	Strengths	Limitations	Key Added Value for Bulgaria
SAQ	Disease-specific (angina)	Physical Limitation, Angina Stability, Angina Frequency, Treatment Satisfaction, QoL	High sensitivity to angina changes; strong prognostic validity; trial-proven responsiveness	Requires translation/cultural adaptation; limited local cost-effectiveness data	Most appropriate for CCS; captures angina-specific burden and treatment response
SAQ-7	Short form (angina)	7-item subset covering key domains	Reduced patient burden; retains validity and prognostic power	Less granular detail; fewer items on stability and satisfaction	Feasible for outpatient centers and registries
SF-36	Generic HRQoL	8 physical/mental/social domains	Widely validated; allows for cross-disease comparability	Less sensitive to angina-specific change; takes longer to complete	Useful for population surveys and comparisons with non-CVD cohorts
EQ-5D	Generic HRQoL	5 domains + VAS; utility scores	Generates QALYs; central for health economic evaluations	Very limited disease-specific sensitivity	Suitable for policy-level analyses and cost–utility studies
MacNew	Disease-specific (cardiac)	Physical, Emotional, Social	Captures psychosocial burden in IHD	Does not assess angina frequency or treatment satisfaction	Could be complementary in psychosocial research
MLHFQ	Disease-specific (HF)	Physical, Emotional	Validated in HF populations	Minimal relevance to CCS	Not recommended as primary tool for CCS

**Table 3:** Major clinical trials and registries incorporating the Seattle Angina Questionnaire (SAQ) as an endpoint.

Trial/Registry	Population	Intervention	Key SAQ Findings	Reference
COURAGE (2007–2008)	2287 patients with stable CAD	PCI + OMT vs. OMT alone	PCI improved SAQ Angina Frequency and QoL domains, though no survival benefit was observed	[10]
ISCHEMIA (2020)	5179 patients with moderate-to-severe ischemia	Initial invasive strategy vs. conservative strategy	Greater SAQ improvements in patients with frequent baseline angina; minimal benefit in those with rare angina	[12]
EuroCTO (2018)	396 patients with CTO	CTO PCI vs. OMT	Successful CTO PCI significantly improved SAQ Angina Frequency, Physical Limitation, and QoL domains	[13]
PREMIER Registry (2009)	MI survivors, USA	Routine clinical management	Lower QoL and dyspnea strongly associated with higher mortality and rehospitalization within 1 year	[17]
TRIUMPH Registry (2012)	Acute MI survivors, multicenter	Routine clinical management	SAQ domains predictive of readmission, functional recovery, and long-term outcomes	[18]
CONCORDANCE Registry (2015)	ACS survivors, Australia	Routine clinical management	Lower SAQ scores independently associated with reduced QoL and higher event rates	[19]

Abbreviations: SAQ—Seattle Angina Questionnaire; CAD—coronary artery disease; PCI—percutaneous coronary intervention; OMT—optimal medical therapy; CTO—chronic total occlusion; QoL—quality of life.

**Table 4:** Diagnostic phenotyping matrix.

Clinical Profile	Imaging	Invasive Indices	Likely Endotype	Next Step
Angina + non-obstructive CCTA	Stress CMR ± ischemia	Abnormal CFR/IMR or microvascular spasm to ACh	MVA	Beta-blocker/ACEi/Statin ± ranolazine
Nocturnal/rest angina, transient ST elevation	Often normal	Epicardial spasm to ACh	VSA	CCB ± long-acting nitrate; avoid non-selective BB
Limiting exertional angina + focal stenosis	Ischemia in target territory	Abnormal FFR/iFR	Obstructive CAD	GDMT ± PCI/CABG

**Table 5:** Endotype → therapy map and expected SAQ response window.

Endotype	Primary Therapy	Adjunctive Therapy	Expected SAQ Response Window
Obstructive CAD	GDMT ± PCI/CABG	Secondary prevention	4–8 weeks after PCI or med titration
Microvascular angina (MVA)	Beta-blocker, ACEi, statin	Ranolazine, trimetazidine	6–12 weeks
Vasospastic angina (VSA)	CCB, nitrates	Nicorandil, statins	2–6 weeks
Mixed/overlap	Combination tailored to dominant mechanism	As per response	Variable (needs serial SAQ)

## Data Availability

No new data were created or analyzed in this study.

## Abbreviations

ACS	Acute Coronary Syndrome
ANOCA	Angina with Non-Obstructive Coronary Arteries
CABG	Coronary Artery Bypass Grafting
CAD	Coronary Artery Disease
CCD	Chronic Coronary Disease (AHA/ACC terminology)
CCS	Chronic Coronary Syndrome (ESC terminology)
CTO	Chronic Total Occlusion
EHR	Electronic Health Record
ESC	European Society of Cardiology
EQ-5D	EuroQol 5-Dimension Questionnaire
HF	Heart Failure
HRQoL	Health-Related Quality of Life
ICC	Intraclass Correlation Coefficient
INOCA	Ischemia with Non-Obstructive Coronary Arteries
LFU	Lost to Follow-Up
MI	Myocardial Infarction
MLHFQ	Minnesota Living with Heart Failure Questionnaire
NSI	National Statistical Institute (Bulgaria)
OMT	Optimal Medical Therapy
PCI	Percutaneous Coronary Intervention
PRO	Patient-Reported Outcome
PROM	Patient-Reported Outcome Measure
QoL	Quality of Life
RCT	Randomized Controlled Trial
SAQ	Seattle Angina Questionnaire
SF-36	36-Item Short Form Health Survey (MOS SF-36)
SIHD	Stable Ischemic Heart Disease
SRM	Standardized Response Mean
